# Aging and leukemic evolution of hematopoietic stem cells under various stress conditions

**DOI:** 10.1186/s41232-020-00138-3

**Published:** 2020-11-05

**Authors:** Shuhei Kurosawa, Atsushi Iwama

**Affiliations:** Division of Stem Cell and Molecular Medicine, Center for Stem Cell Biology and Regenerative Medicine, The Institute of Medical Science, The University of Tokyo, 4-6-1 Shirokanedai, Minato-ku, Tokyo, 108-8639 Japan

**Keywords:** Hematopoietic stem cell, Aging, Reactive oxygen species, DNA damage, Polarity, Stem cell niche, Senescence, Epigenetics, Age-related clonal hematopoiesis, Clonal hematopoiesis of indeterminate potential

## Abstract

Hematopoietic stem cells (HSCs) have self-renewal capacity and differentiation potential into all lineages of blood cells throughout the lifetime of an organism. The function of HSCs gradually changes during aging. To date, various stress factors influencing HSC aging have been identified. The increased production of reactive oxygen species and DNA damage responses are causatively attributed to HSC aging. The increased apolarity is a prominent feature of aged HSCs, whereas it is less obvious in young HSCs. The bone marrow (BM) microenvironment niche is a crucial factor for HSC aging. Mesenchymal stem cells show skewed differentiation during aging, which leads to decreased bone formation and increased adipogenesis. The accumulation of adipocytes confers negative effects on hematopoiesis. Loss of sympathetic nerve fibers or adrenoreceptor β3 signaling induces premature HSC and niche aging. Epigenetic regulators such as polycomb group proteins and the sirtuin family of proteins act to prevent premature aging. Targeting these factors, several rejuvenation strategies for aged HSCs have been employed in mice. However, we still do not know whether these strategies can be extrapolated to human HSCs. Aging is frequently accompanied by the development of clonal hematopoiesis, which is called age-related clonal hematopoiesis (ARCH) or clonal hematopoiesis of indeterminate potential (CHIP). Most ARCH/CHIP mutations occur in genes encoding epigenetic regulators including *DNMT3A*, *TET2*, and *ASXL1*, which suggests the relevance of epigenetic drift during the aging process. ARCH/CHIP is a strong risk factor for subsequent hematologic cancer. Notably, it also has an impact on the development of non-malignant disorders such as coronary heart disease. Further studies are warranted to decipher the complete picture of molecular crosstalk that regulates HSC aging.

## Background

Hematopoietic stem cells (HSCs) are characterized by their ability to self-renew and their potential to differentiate into all lineages of blood cells throughout the lifetime of an organism. During aging, the functionality of HSCs gradually changes in mice as well as humans. Aged HSCs show several characteristic alterations in phenotype, such as an increase in the frequency of immunophenotypic HSCs, a decrease in regenerative capacity [1–3], myeloid-biased differentiation [1, 2], impaired homing and engraftment upon transplantation [4], platelet-biased differentiation [5], and megakaryocytic/erythroid-biased gene expression patterns [6]. Additionally, in humans, age-associated hematopoietic changes are correlated with an increased incidence of myeloid malignancies [7, 8]. In this review, we summarize the current knowledge on the mechanisms of HSC aging and clonal hematopoiesis, as well as the results of recent single-cell profiling studies on aged HSCs.

## Main text

### Factors contributing to HSC aging

**Fig. 1:**
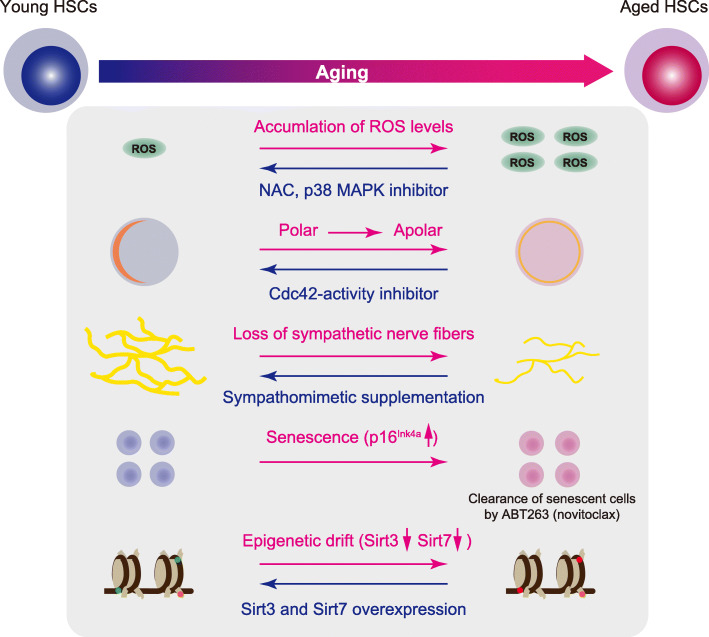
Factors that promote HSC aging and rejuvenation strategies for aged HSCs. *N*-acetyl cysteine (NAC) and p38 mitogen-activated protein kinase (MAPK) inhibitor rejuvenate aged hematopoietic stem cells (HSCs) by reducing reactive oxygen species (ROS) levels. Cdc42 activity inhibitor (CASIN) increases the percentage of polarized cells, restores the spatial distribution of H4 at lysine 16 acetylation, increases lymphoid output, and reduces myeloid lineage output. Sirt3 overexpression restores the long-term competitive repopulation ability. Sirt7 overexpression rescues myeloid-biased differentiation. ABT263 (navitoclax), a BCL-2- and BCL-xL-specific inhibitor, selectively kills senescent cells. Supplementation of a sympathomimetic acting selectively on adrenoreceptor β3 rejuvenates the function of aged HSCs. The red and blue arrows indicate aging and rejuvenation, respectively

Over the past decade, the multiple differences between young and aged HSCs have been examined, and various stress factors influencing HSC aging have been identified [8–13]. Functional decline in aged HSCs is associated with changes in cell-intrinsic aging drivers, such as metabolic alternations, impaired autophagy, proteostasis, DNA damage, senescence, and other signaling pathways (mTOR, WNT, Janus kinase, and signal transducer and activator of transcription, NF-κB, and TGF-β). Loss of epigenetic regulation (DNA methylation, histone marks, and chromatin architecture) and cell polarity are additional cell-intrinsic features. Additionally, cell-extrinsic factors, including the development of a proinflammatory milieu and decreased function of the old bone marrow (BM) niche, contribute to HSC aging. Here, we focus on the current understanding of these factors, particularly reactive oxygen species (ROS), DNA damage, polarity, stem cell niches, senescence, and epigenetics (Fig. 1).

#### ROS

The maintenance of appropriate ROS levels is critical for the maintenance of HSCs and DNA integrity, as well as for preventing ROS-mediated cell damage [14]. Jang and Sharkis reported that HSCs with low levels of ROS had high potential for self-renewal, which was attenuated during aging as the ROS levels increased [15]. A ROS inhibitor—*N*-acetyl cysteine (NAC)—and a p38 mitogen-activated protein kinase (MAPK) inhibitor have been shown to rescue the decreased functionality of HSCs with high levels of ROS, demonstrating a role of ROS and p38 MAPK in ROS-mediated HSC maintenance.

Forkhead O (FOXO) family transcription factors, which are important downstream targets of the PI3K-AKT pathway, play a significant role in mediating the response to physiological oxidative stress and are essential for the self-renewal of HSCs. The HSCs of mice deficient in Foxo1, Foxo3a, and Foxo4a show a marked increase in ROS levels due to the dysregulation of genes that regulate ROS [16]. Miyamoto et al. generated gene-targeted Foxo3a^−/−^ mice and showed that HSC frequencies were significantly decreased in aged Foxo3a^−/−^ mice than in the littermate controls [17].

#### DNA damage

DNA damage is caused by various factors, such as radiation, exogenous genotoxins, and free radicals, and has been observed to accumulate during aging in many studies [18–20]. Phosphorylation of histone H2AX (γ-H2AX) is an indicator of DNA damage, and HSCs accumulate γ-H2AX signals with age [21]. Older HSCs which are still undergoing the cell cycle show heightened levels of replication-associated γ-H2AX foci and increased levels of replication stress associated with cell cycle defects, altered DNA fork replication dynamics, and chromosome gaps or breaks [22]. Nonetheless, once old HSCs re-establish quiescence, residual replication stress on ribosomal DNA genes leads to the formation of nucleolar-associated γH2AX signals, which persist owing to ineffective H2AX dephosphorylation rather than ongoing DNA damage. Thus, persistent nucleolar γH2AX acts as a histone modification, marking the transcriptional silencing of rDNA genes and decreased ribosome biogenesis in quiescent old HSCs.

The DNA damage response is mainly regulated by the following pathways: base excision repair, nucleotide excision repair (NER), homologous recombination repair (HR), and non-homologous end joining repair (NHEJ) [21–25]. The NER pathway maintains HSC functionality during aging by preserving the cells’ ability for reconstitution, self-renewal, and proliferation, and by preventing programmed cell death under conditions of stress [21]. The NER pathway-associated gene Xab2 has been reported to be downregulated in aged HSCs, suggesting that the NER pathway acts to rescue HSC function, but its activity decreases during aging [23]. Further analysis revealed that quiescent hematopoietic stem and progenitor cells (HSPCs) undergo error-prone NHEJ-mediated DNA repair while proliferating HSPCs do HR-mediated DNA repair [24]. HR-mediated DNA repair uses a template for accurate repair, typically the sister chromatid, and thus can only occur in cycling cells. In contrast, NHEJ-mediated DNA repair has a limited requirement for sequence homology and can occur during any stage of the cell cycle [25]. The accumulation of NHEJ-mediated mutations impairs HSC performance and is a major factor in the loss of function observed in aged HSCs.

#### Polarity and WNT signaling

A reduction in the number of cells with a polar distribution of microtubules has been observed in aged early hematopoietic progenitor cells [26]. Florian et al. reported that elevated activity of the small Rho GTPase Cdc42 is correlated with the loss of polarity in aged HSCs [27]. A Cdc42 activity inhibitor (CASIN) rejuvenated the functionality of aged HSCs, improved the percentage of polarized cells in aged HSCs, and restored the level and spatial distribution of histone H4 at lysine 16 acetylation (H4K16ac), which has been found to be reduced in multiple tissues in aged mice.

The WNT pathway proteins function as proliferation-inducing growth factors and affect cell-fate decisions, apoptosis, and quiescence [28]. WNT3a—which is associated with the canonical pathway—and WNT5a—which is associated with the non-canonical pathway—are the most widely studied proteins in hematopoiesis. The former promotes lymphopoiesis, and the latter enhances myelopoiesis [29]. Florian et al. reported that elevated expression of Wnt5a in aged HSCs results in apolarity through increased Cdc42 activity [30]. The activation of Cdc42 by Wnt5a treatment-induced aging-related stem cell apolarity, reduction of the self-renewal potential, and myeloid-skewed differentiation in young HSCs. Povinelli and Nemeth reported that inhibition of the Wnt ligand receptor Ryk blocked the ability of WNT5a to induce HSC quiescence, and enhanced short- and long-term hematopoietic repopulation through reduction of the intracellular levels of ROS [31].

#### Stem cell niches

Niche factors secreted from aging stromal cells play a critical role in shifting the subtype of HSCs. RANTES (CCL5) is a key proinflammatory cytokine that accumulates in aged BM and is expressed by stromal as well as differentiated blood cells [32]. Ergen et al. reported that a brief ex vivo exposure of HSCs to Rantes resulted in T cell deficiency and expanded the myeloid progenitors. In contrast, the deletion of Rantes in mice rescued myeloid-biased differentiation and increased T cell production [33].

An additional hallmark of aging is altered HSC distribution. Aging induces significant remodeling of HSC niches. Mesenchymal stem cells exhibit skewed differentiation during aging, leading to decreased bone formation and increased adipogenesis [34, 35]. The accumulation of adipocytes in the BM negatively affects hematopoiesis [36–38]. Arterioles and transitional (type H) vessels that connect to the arterioles are found in direct proximity and share many features that distinguish them from BM sinusoidal (type L) capillaries. Type H capillaries are surrounded by osteoprogenitors, and both cells as well as arterioles decline significantly with aging [39, 40]. Activation of endothelial Notch signaling can restore the numbers of arteries and type H capillaries, although HSC function is not rejuvenated [40]. Aged mice also show a significant increase in vascular leakiness and ROS levels in endothelial cells in BM [41].

The sympathetic nervous system (SNS) regulates the attraction of stem cells to the microenvironment by orchestrating the release of adrenergic neurotransmitter in a circadian manner [42, 43]. Maryanovich et al. reported that loss of sympathetic nerves around the arteriolar niche, or adrenoreceptor β3 signaling, induced the premature aging of HSCs and niche, indicating that BM innervation by the SNS regulates the aging of HSCs and their niche [44]. Supplementation of a sympathomimetic acting selectively on the adrenoreceptor β3 of aged mice significantly rejuvenated the function of aged HSCs.

#### Senescence

Senescent cells show a characteristic phenotype known as the senescence-associated secretory phenotype (SASP), in which the cells secrete high levels of proinflammatory and matrix-degrading molecules [45]. Two major pathways are implicated in the induction of senescence, tumor suppressors p16^Ink4a^ and p19^Arf^ (p14^ARF^ in humans) that are encoded by the genes at the Ink4a/Arf (Cdkn2a) locus [46]. p19^Arf^ stabilizes p53 by inhibiting MDM2, resulting in the activation of p53 target genes involved in cell cycle arrest and apoptosis. p16^Ink4a^ blocks the assembly of catalytically active cyclin D-CDK4/6 complexes by maintaining Rb in a hypophosphorylated state. p16^Ink4a^ expression in HSCs increases with age, and the absence of p16^Ink4a^ could mitigate the repopulating defects in HSCs [47]. Chang et al. reported that ABT263 (navitoclax), a BCL-2- and BCL-xL-specific inhibitor, selectively induces the apoptosis of senescent HSCs after total body irradiation (TBI), mitigating TBI-induced premature aging of the hematopoietic system, and rejuvenating aged HSCs in normally aged mice [48].

### Epigenetics

Stem cells have developed epigenetic regulatory mechanisms to prevent premature aging [49]. Polycomb group (PcG) proteins are representative of such regulators. The PcG protein complexes repress the expression of their target genes and are divided into the polycomb repressive complex 1 (PRC1) and PRC2. The former mono-ubiquitinates histone H2A at lysine 119 (H2AK119Ub), and the latter trimethylates histone H3 at lysine 27 (H3K27me3) [50]. PcG complexes target the Cdkn2a locus and regulate HSCs by acting as critical failsafe against the premature aging induced by the p16^Ink4a^ and p19^Arf^ tumor suppressors [51–55]. Forced expression of PcG genes, such as Ezh2, Kdm2b/Fbxl10, Bmi1, and Cbx7, promotes HSC self-renewal and preserves HSC potential during serial transplantations, an observation which is also indicative of their role in HSC aging [56–60]. It remains to be investigated how changes in these genes contribute to HSC aging other than by their impact on the Ink4a/Arf locus.

Members of the sirtuin family of proteins are NAD^+^-dependent deacetylases, which play key roles in responding to nutritional and environmental perturbations such as DNA damage and oxidative stress [61]. Mammals have seven sirtuins, SIRT1–7: SIRT1, SIRT6, and SIRT7 in the nucleus; SIRT3, SIRT4, and SIRT5 in the mitochondria; and SIRT2 in the cytoplasm [62]. In HSPCs, Sirt1 ablation increases Hoxa9 expression and HSPC expansion, depending on hematopoietic stress [63]. However, it causes genomic instability as well as the accumulation of DNA damage and eventually results in a loss of long-term HSPCs.

Sirt3 deacetylates and activates mitochondrial isocitrate dehydrogenase 2, leading to increased levels of NADPH and an increased ratio of reduced-to-oxidized glutathione in the mitochondria [64]. The levels of Sirt3 are reduced during aging, and its overexpression rescues functional defects in aged HSCs in mice [65]. Sirt7 expression is also reduced in aged HSCs, while its overexpression increases the reconstitution capacity of HSCs and attenuates myeloid-biased differentiation in aged HSCs [66]. This effect is probably mediated by the mitochondrial unfolded protein response, a signaling pathway that regulates mitochondrial chaperone transcription and is critical for stress relief.

### Age-related clonal hematopoiesis/clonal hematopoiesis of indeterminate potential

**Fig. 2:**
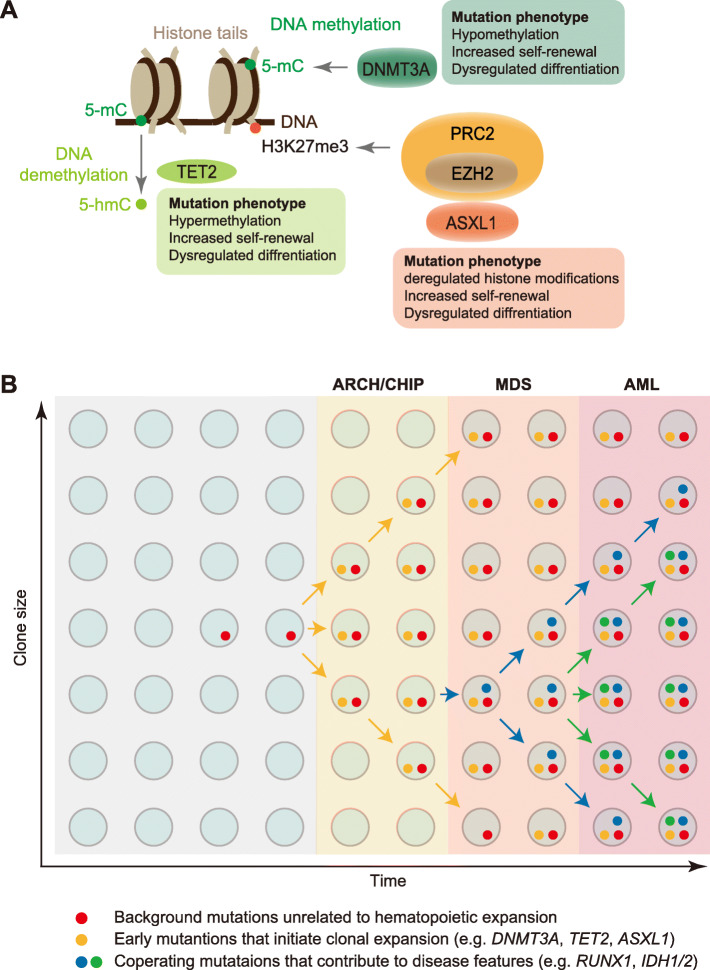
Epigenetic regulators of clonal hematopoiesis. **a** Schematic representation of major epigenetic regulators involved in clonal hematopoiesis and age-associated myeloid malignancies. Mutation phenotypes of *DNMT3A*, *TET2*, and *ASXL1* are summarized. 5-mC, 5-methylcytosine; 5-hmC, 5-hydroxymethylcytosine; H3K27me3, trimethylated H3 at lysine 27. **b** Age-associated clonal hematopoiesis. Age-related clonal hematopoiesis (ARCH)/clonal hematopoiesis of indeterminate potential (CHIP) is asymptomatic clonal hematopoiesis characterized mainly by mutations in *TET2*, *DNMT3A*, or *ASXL1*. ARCH/CHIP may progress with additional mutations such as *RUNX1*, *EZH2*, and *IDH1/2* to MDS and ultimately AML. ARCH/CHIP may also progress directly to AML without an intervening MDS stage or to other conditions such as myeloproliferative neoplasms or lymphoid neoplasms. AML, acute myeloid leukemia; MDS, myelodysplastic syndrome; PRC, polycomb repressive complex

Recently, several groups have used next-generation genome sequencing to identify recurrent somatic mutations with clonal hematopoiesis in healthy elderly individuals [67–69]. This phenomenon is called age-related clonal hematopoiesis (ARCH) or clonal hematopoiesis of indeterminate potential (CHIP) [70, 71]. ARCH/CHIP determined using peripheral blood is extremely rare in young individuals (< 1% in persons < 40 years of age) but increases exponentially with age. These mutations are observed in 9.5% of the individuals aged 70 to 79, 11.7% of those aged 80 to 89, and 18.4% of those over 90 [68]. ARCH/CHIP is mainly associated with mutations in three epigenetic regulatory genes—*DNMT3A*, *TET2*, and *ASXL1*—that have been implicated in hematologic cancers and is a strong risk factor for subsequent hematological cancers [69] (Fig. 2a). Mutant HSCs in this spectrum acquire additional mutations that can lead to a disease phenotype and ultimately morbidity and mortality. Upon serial acquisition of mutations, ARCH/CHIP can progress to myelodysplastic syndrome (MDS) and ultimately acute myeloid leukemia (AML), directly to AML without an intervening MDS stage, or to other conditions such as myeloproliferative neoplasms (MPN) or lymphoid neoplasms [70, 72]. In addition to ARCH/CHIP, *DNMT3A*, *TET2*, and *ASXL1* mutations are frequently detected in several myeloid malignancies, including MDS, MDS/MPN, and AML [73–75], suggesting that these mutations are the earliest events during malignant transformation.

DNMT3A is a member of a family of DNA methyltransferases that catalyzes DNA methylation [76]. Loss-of-function mutations in *Dnmt3a* augment the self-renewal capacity of HSCs and cause myeloid-biased differentiation, leading to a fitness advantage in HSC clones in mice [77, 78]. Analysis of large adult AML cohorts revealed *DNMT3A* mutations frequently co-occurring with *FLT3*, *NRAS*, *KRAS*, *PTPN11*, and *NPM1* [73, 74]. Experimental studies in mice confirmed that Dnmt3a loss synergized with an active Nras mutant, leading to the rapid development of leukemia [79]. Similar studies were performed with mutant Flt3 overexpression, which was shown to lead to the development of both myeloid and lymphoid leukemias [80].

TET2 is involved in DNA demethylation pathways converting 5-methylcytosine to 5-hydroxymethylcytosine (5-hmC), 5-formylcytosine, and 5-carboxylcytosine [81]. *TET2* loss-of-function mutations are associated with hypermethylation. Mouse studies of conditional Tet2 loss revealed expansion of Lineage^-^Sca1^+^cKit^+^ cells concomitant with decreased 5-hmC levels [82, 83]. *Tet2*-mutant mice have been shown to synergize with *Flt3*-mutant mice, leading to the development of AML with full penetrance [84]. *TET2* mutations frequently co-occur with loss-of-function *EZH2* mutations in humans [85, 86]. We examined the impacts of loss-of-function *EZH2* mutations on the pathogenesis of myeloid malignancies using *Ezh2* conditional knockout mice [87] and demonstrated that an *Ezh2* deficiency in combination with a *Tet2* hypomorph in mice accelerates the transformation of HSCs and induces MDS and MDS/MPN [88].

ASXL1 is involved in mediating a number of histone modifications, such as H3K27me3, H2AK119Ub, and histone H3 at lysine 4 trimethylation (H3K4me3), which regulate gene expression, and might function as a scaffold for epigenetic regulators [89]. Loss of ASXL1 results in the global exclusion of H3K27me3, indicating that ASXL1 cooperates with PRC2 to regulate H3K27me3 [90]. ASXL1 forms a complex with the deubiquitination enzyme BAP1 and removes monoubiquitin from H2AK119Ub, to derepress genes targeted by PRC1 [91]. Recent studies using mice expressing an *ASXL1* mutant demonstrated that an *ASXL1* mutation alone is not sufficient for inducing the development of hematologic malignancies [92–94]. However, an *ASXL1* mutation increased the susceptibility to leukemogenesis in concert with a *RUNX1* mutant or in viral insertional mutagenesis, indicating that mice expressing an *ASXL1* mutant represent a premalignant condition like ARCH/CHIP [93].

ARCH/CHIP progresses under selection pressure such as that imposed by aging, chemotherapy, or immune-mediated clonal selection [95]. Mutations in *DNMT3A*, *TET2*, and *ASXL1* appear to offer a selective advantage to HSC clones over non-mutated clones by maximizing self-renewal and modulating differentiation, suggesting that a dysregulated epigenome increases the epigenetic heterogeneity that eventually leads to the appearance of HSC clones with better fitness in the aged BM niche. Recent studies have shown that chronic infection depletes normal HSCs and multipotent progenitor cells in an interferon γ-dependent manner [96] and that elevated levels of TNF selectively favor the expansion of *TET2*-mutant HSCs [97]. Moreover, Tet2 recruits Hdac2 and represses the transcription of *Il6* via histone deacetylation [98]. Under inflammatory stress, *Tet2*-deficient HSCs resist apoptosis, rapidly expand, and produce more proinflammatory cytokines, including IL-6 [99]. These findings suggest that inflammation and inflammatory aged BM microenvironment enhance the clonal expansion of HSCs with driver mutations over normal aged HSCs [100].

As we described above, the co-occurrence of mutations is important for the progression from ARCH/CHIP to MDS and AML [79, 80, 84, 88, 93]. Additionally, the order of mutation acquisition may be crucial for disease pathogenesis (Fig. 2b) [72]. Targeted mutational analysis of patients with de novo and secondary AML revealed that mutations in *SRSF2*, *SF3B1*, *U2AF1*, *ZRSR2*, *ASXL1*, *EZH2*, *BCOR*, or *STAG2* were highly specific for secondary AML and occurred early in leukemogenesis [101]. Makishima et al. identified two classes of mutated genes by sequencing MDS and secondary AML samples: type 1 enriched in secondary AML compared with high-risk MDS (*FLT3*, *PTPN11*, *WT1*, *IDH1*, *NPM1*, *IDH2*, and *NRAS*) and type 2 enriched in high-risk compared with low-risk MDS (*TP53*, *GATA2*, *KRAS*, *RUNX1*, *STAG2*, *ASXL1*, *ZRSR2*, and *TET2*) [102]. Another sequencing study of serial samples from patients with secondary AML revealed that mutations in genes related to DNA methylation and splicing machinery were early disease events of myelodysplasia [103]. In contrast, somatic variants associated with signaling pathways arose or their allelic burdens expanded during progression. Further studies of a larger number of serial samples may improve the understanding of the order of mutation acquisition.

Continuous follow-up is important for patients diagnosed with ARCH/CHIP. ARCH/CHIP can be transferred from donor to recipient during allogeneic HSC transplantation. Gondek et al. reported two cases of donor cell leukemia arising from ARCH/CHIP marked by somatic mutations in leukemia-related genes in donors [104]. This study suggests that the recognition and diagnosis of ARCH/CHIP are important before the assessment and selection of a donor, particularly for donors older than 50–60 years [105]. In contrast, screening for ARCH/CHIP among potential donors may have several ethical and operational implications related to disclosure of the results [106]. The current level of evidence is insufficient to support the implementation of prospective screening of donors for ARCH/CHIP. Further studies may reveal the magnitude of risk or benefit associated with screening for ARCH/CHIP.

ARCH/CHIP also has an impact on the development of non-malignant diseases. Coronary heart disease has been observed to occur 1.9 times more frequently in individuals with ARCH/CHIP than in those without ARCH/CHIP [107]. Although the mechanism underlying the association of ARCH/CHIP with coronary heart disease remains to be investigated, driver mutations such as *TET2* and *DNMT3A* have been reported to cause phenotypic changes in HSCs and immune cells, including increased inflammatory responses in macrophages and mast cells, and functional alterations in T cells [107–109].

### Application of single-cell technologies to HSC aging

Recently, single-cell analysis has become a powerful tool for studying cellular differentiation pathways [110]. These advances in technology have made it possible to reveal differences intrinsic to the cell during the aging of HSCs. Grover et al. analyzed old and young HSC transcriptomes at the single-cell level and found that platelet-biased HSCs preferentially expand during aging, and aged HSCs are functionally platelet-biased [5]. They observed that loss of the FOG-1 transcription factor that is associated with HSC platelet priming increases lymphoid output, suggesting that increased platelet bias contributes to the age-associated decrease in lymphopoiesis. Yamamoto et al. performed large-scale clonal analysis using single HSC transplantation and found that the frequency of myeloid-restricted repopulating progenitors increases dramatically with age, while multipotent HSCs expand modestly in the BM [111]. They also identified a subset of functional HSCs that are myeloid-restricted in primary recipients but display multi-lineage outputs in secondary recipients.

Single-cell epigenomic technologies such as single-cell chromatin immunoprecipitation sequencing and single-cell assays for transposase-accessible chromatin (ATAC) sequencing have been employed to investigate histone modifications and to map the accessible chromatin regions, respectively [112]. Florian et al. performed a comprehensive set of paired daughter cell analyses that included single-cell 3D confocal imaging, single-cell transplants, single-cell RNA-seq, and single-cell ATAC-seq. They found that polar HSCs (that are the major cells in young mice) preferentially sort the small RhoGTPase Cdc42 asymmetrically while apolar HSCs (that are the majority in aged mice) sort symmetrically during cell division [113]. These findings suggest that the outcome of HSC division is strongly linked to the polarity status before mitosis, which is in turn determined by Cdc42 activity in HSCs. They also showed that the hematopoietic potential of daughter cells is linked to the amount of the epigenetic mark H4K16ac and to the amount of open chromatin allocated to a daughter cell, but not to its transcriptome. Cheung et al. employed a highly multiplexed mass cytometric analysis to profile the global levels of a wide array of chromatin modifications in immune cells at the single-cell level and found markedly different cell type- and hematopoietic lineage-specific chromatin modification patterns [114]. Differential analysis of HSPCs from younger and older adults revealed that aging was associated with increased heterogeneity between individuals and elevated cell-to-cell variability in chromatin modifications.

## Conclusions

In this review, we have described various stresses that promote HSC aging and clonal hematopoiesis with age. We also discussed recent studies on HSC aging using single-cell technologies at both the transcriptomic and epigenetic levels. There is no doubt that HSCs show declining function during aging, but we still do not know whether this dysfunction is reversible in humans. Some promising strategies have been employed to rejuvenate aged HSCs based on stress factors intrinsic and extrinsic to the cell, including NAC and p38 MAPK inhibitor [16], CASIN [28], epigenetic manipulation such as Sirt3 and Sirt7 overexpression [65, 66], sympathomimetic supplementation [44], and ABT263 (navitoclax) [48] (Fig. 1). In humans, ABT263 has already been used, but its use has been limited by on-target thrombocytopenia [115, 116]. Nevertheless, such interventions may be adapted for treating HSC aging in humans with acceptable toxicity through new approaches in the formulation, delivery, or administration schedules.

Although the aging of HSCs has been extensively studied, there are some conflicting results. Therefore, we should carefully consider the differences in systems and experimental methods between these studies. We also need to examine how findings obtained from mice with 2-year lifespans can be extrapolated to human HSCs. The elderly population is rapidly increasing worldwide. Basic and clinical studies are needed to elucidate aging-related hematopoietic pathology to improve the clinical outcome of patients with these pathologies.

## Data Availability

Not applicable

## Abbreviations

AML: Acute myeloid leukemia
ARCH: Age-related clonal hematopoiesis
ATAC: Assays for transposase-accessible chromatin
BM: Bone marrow
CHIP: Clonal hematopoiesis of indeterminate potential
H2AK119Ub: H2A at lysine 119 ubiquitination
H3K4me3: H3 at lysine 4 trimethylation
H3K27me3: H3 at lysine 27 trimethylation
H4K16ac: H4 at lysine 16 acetylation
HSCs: Hematopoietic stem cells
HSPCs: Hematopoietic stem and progenitor cells
MAPK: Mitogen-activated protein kinase
MDS: Myelodysplastic syndrome
MPN: Myeloproliferative neoplasms
NAC: *N*-acetyl cysteine
NER: Nucleotide excision repair
NHEJ: Non-homologous end joining
PcG: Polycomb group
PRC: Polycomb repressive complex
ROS: Reactive oxygen species
SASP: Senescence-associated secretory phenotype
SNS: Sympathetic nervous system
